# Not by the Book: Observations of Delayed Oviposition and Re-Colonization of Human Remains by Blow Flies

**DOI:** 10.3390/insects13100879

**Published:** 2022-09-28

**Authors:** Charity G. Owings, Hayden S. McKee-Zech, Sarah T. Schwing, Kristi N. Bugajski, Mary C. Davis, Dawnie W. Steadman

**Affiliations:** 1 Department of Anthropology, University of Tennessee, 1621 Cumberland Ave., Knoxville, TN 37996, USA; 2 Department of Biology, Valparaiso University, 1610 Campus Drive East, Valparaiso, IN 46383, USA

**Keywords:** blow fly, time of colonization, decomposition, total body score

## Abstract

**Simple Summary:**

Atypical blow fly colonization is rarely documented in the forensic entomology literature. We demonstrate one case of an exaggerated delay in blow fly colonization and two cases of blow fly re-colonization after cessation of the consumption phase on human remains at the Anthropology Research Facility (University of Tennessee). We recommend interdisciplinary collaboration to better interpret evidence encountered from scenarios comparable to these examples.

**Abstract:**

Postmortem interval estimations can be complicated by the inter-individual variation present in human decomposition. Forensic entomologists may especially face challenges interpreting arthropod evidence in scenarios that are not “by the book”, or that vary in unexpected ways. Therefore, it is important to report instances where blow fly colonization does not align with expected soft tissue decomposition as blow fly larvae are often used to produce a time of colonization (TOC) estimation to infer a minimum PMI. We followed the decomposition and blow fly activity of three human donors at the Anthropology Research Facility (University of Tennessee). Delayed oviposition occurred on one donor 115 d post-placement, whereas two donors experienced blow fly re-colonization after cessation of the consumption phase, one 22 d and one more than 200 d after blow fly larvae were last observed. A null hypothesis model tested whether the entomological TOC and anthropological total body score (TBS) estimations encompassed the time of placement (TOP) for each donor. While the null hypothesis was rejected for all TOC estimations, it could not be rejected for the TBS estimations. We discuss how the non-linear nature of human decomposition can pose challenges to interpreting blow fly evidence and suggest that forensic entomology practitioners should recognize these limitations in both research endeavors and applied casework.

## 1. Introduction

A common objective across many forensic science disciplines is the determination of the postmortem interval (PMI), or the time period beginning at an individual’s death and ending at the discovery of their remains [1,2,3]. The field of forensic entomology encompasses the study of arthropods associated with civil and criminal legal investigations [4], and several decades of foundational research in this field have demonstrated that arthropods can be powerful indicators of portions of the PMI, given that certain assumptions are met [5,6,7,8]. Forensic entomologists rely on aspects of arthropod biology to form such an estimation, including utilizing succession analyses [9] or the growth rate of immature arthropods found on the decedent [10]. However, such estimations ultimately rely on life history traits and behaviors that can be significantly affected by environmental conditions [11,12], potentially impacting the accuracy of these methods.

Succession models, which utilize the semi-predictable chronological changes in arthropod community composition on vertebrate remains, should represent the best, most accurate method available for estimating the entire PMI as these studies automatically incorporate the time of death (TOD) when scoring the presence or absence of different taxa and life stages on the remains [13,14]. However, the utility of this method is limited by the variability in arthropod community composition across spatial, temporal, and seasonal scales, as well as by the population dynamics of any given species in the target community [15]. Additionally, an overall lack of design standardization across studies prevents useful comparisons and validation of datasets [16,17]. The second method uses an age determination (based on published, species-specific growth rate data) of immature arthropods to estimate a possible window of time for which individuals in a given sample could have begun their life cycle on the decedent [18]. This window of time is known as the time of colonization (TOC). The TOC estimation can then be used by investigators to infer a portion of the PMI, known as the minimum PMI (PMI_MIN_ or mPMI) [5]. Though the PMI and PMI_MIN_ are sometimes used interchangeably, the most significant difference is that the PMI begins at the TOD, whereas the PMI_MIN_ begins at the TOC [19]. Though it is possible that the PMI_MIN_ = PMI (i.e., the TOC = TOD) under some circumstances, it is often impossible to know if this is true in forensic casework [20]. Thorough comparisons and visual illustrations of these definitions have previously been reviewed [8,19]. Similar to the succession method, the TOC method is also not immune to error, as it is only useful when sufficient biological information is known for a given species. Multiple factors can contribute to the inaccuracy of age estimations, such as the true temperature experienced by larvae feeding on remains [21,22,23,24,25] (including neglecting the species-specific thermoregulatory abilities of larvae [26,27]), additional intrinsic and environmental factors that impact larval development rate (e.g., tissue type, density, and genetics [28,29,30,31,32,33,34,35]), and even the developmental datasets used to produce an estimation range [19,36]. As such, forensic entomologists are urged to be transparent about the assumptions being made regarding the generation of entomological estimations in forensic casework [8,37].

A challenge for entomological methods can occur when there is a mismatch between the occurrence of immature blow flies and the current progression of decomposition. Adult blow flies are known to arrive during fresh decomposition to oviposit, with the blow fly larval consumption phase [18] lasting a variable amount of time depending on biotic and abiotic factors (e.g., species, geography, temperature). For example, larval dispersal has been observed by seven days post-placement in the summer in the southern US [38,39], though this time period can be doubled in the summer in the midwestern US [13]. In the spring, blow fly larvae have been recorded as being present up to 50 d post-placement in the southern US [40] and 62 d in Canada [41]. In the winter, blow fly larvae have been found up to 66 d post-placement in the southern US [42]. The presence of blow fly larvae during later decomposition could be attributed to a number of factors, including limited food access (a possible effect of competition [43,44]), poor resource quality (e.g., when tissue becomes desiccated [45] or is saturated with potentially toxic substances [46]), cold temperatures [47], seasonal population distribution [48], and even the sex of larvae (females tend to develop more slowly than males [34]). As decomposition proceeds in these types of situations, greater inaccuracy of entomological estimations is inevitable [49].

In the field of forensic anthropology, practitioners endeavor to make full PMI estimations based on the decomposition progression of the remains, which can be of particular use in late decomposition when tissues begin to mummify. Forensic anthropology is a subfield of biological anthropology that applies osteological and archeological methods, as well as the study of vertebrate decomposition and taphonomy, to forensic death investigations [50]. Forensic anthropologists have developed methods for estimating the PMI based on soft tissue decomposition of human remains. The most widely used method, called the Total Body Score (TBS), consists of correlating soft tissue decomposition changes in three body regions (head and neck, trunk, and limbs) to the time since death recorded in accumulated degree days (ADD) [51,52]. The TBS method is a quasi-quantitative tool used for estimation of the PMI that relies on visual morphological changes and a linear regression model to produce ADD estimates. Using a summed regional point system (the sum being the Total Body Score), the TBS method assumes that decomposition is a semi-continuous variable (rather than one categorizable into a few discrete stages) that is directly correlated with temperature. The TBS method is the most common PMI estimation tool utilized by forensic anthropologists given its ability to be actively or retroactively applied (i.e., to physical remains or photographs of physical remains) and its lack for need of specialized equipment or extensive experience by the user [53,54]. However, due to limitations associated with the taphonomic conditions it can account for (e.g., tissue damage from vertebrate scavenging is not considered) and an error of 388.2 ADD [52], it is by no means a gold standard [51,55,56].

We present three instances of under-reported blow fly colonization patterns on human remains. These cases involve discrepancies between the observed blow fly activity and the expected soft-tissue decomposition of the remains from which specimens were collected. Without context, or the invaluable experience of observing human decomposition in semi-controlled environments (e.g., at human decomposition facilities), non-specialist investigators (i.e., non-entomologists) may misinterpret the value of blow fly evidence found in scenarios similar to those outlined in this paper.

## 2. Materials and Methods

### 2.1. Study Population

All research was performed at the Anthropology Research Facility (ARF), which is an outdoor research facility operated by the Forensic Anthropology Center (FAC) at the University of Tennessee (UTK) in Knoxville, TN, USA. The ARF is a wooded facility in a humid subtropical climate with an average yearly temperature of 15 °C. The FAC maintains a world-class body donation program that accepts approximately 100 human donors per year for various decomposition projects and osteological research [57]. The ARF, which is the first outdoor human decomposition facility and is colloquially known as the “Body Farm”, hosts 200–300 sets of human remains at varying states of decomposition at any given time.

Human donors were not specifically placed for this study. Rather, they were chosen for further examination after their unusual decomposition or colonization patterns became apparent to the authors. All donors used in this study were placed fresh (i.e., unfrozen, experienced cooler storage prior to placement, placed at the ARF 24–48 h after arrival to the FAC) and unclothed on the ground surface in the supine position at the ARF from winter 2019 to spring 2021 (Table 1). No external trauma was noted prior to placement for any of the donors used in this study. All donors were in their early 60s and were considered obese according to the Centers for Disease Control and Prevention [58]. Two donors (Donors 1 and 2) were placed as part of an NIJ-funded study (NIJ # 2018-DU-BX-0180) [46]. The other donor (Donor 3) was placed for general entomology surveys.

### 2.2. Arthropod Observations and Collections

The pre-colonization interval [59] (pre-CI; defined here as the delay between donor placement in the field and first oviposition event by blow flies) was measured in hours for each donor. The NIJ donors (Donors 1 and 2) were observed twice daily from placement until the end of active decomposition (i.e., abdominal collapse and cessation of fluid purge). Observations included detailed photographs, field documentation of arthropod and scavenging activity, documentation of the first colonization event (evidenced by visible eggs), and larval sampling. Larval sampling for Donor 1 did not occur until advanced decomposition due to a delay in initial blow fly oviposition (see Section 3). Additionally, game cameras captured images of vertebrate scavenging for the NIJ donors. Donor 3 was observed daily after placement for initial blow fly colonization and observations of arthropod activity continued weekly for two months. During each observation timepoint for each donor, larvae were collected from each prominent larval aggregation, par-boiled, and preserved in 70% ethanol. All preserved specimens were identified to species using dichotomous keys [60,61,62].

### 2.3. Hypothesis Testing

To determine estimation accuracy, it was critical to choose a known event for which both the TOC and the TBS methods could be compared to in the form of a null hypothesis [8]. Though the TOD was known for the donors in this study, the lag time between death and donor placement at the ARF was highly variable. Therefore, the TOD was excluded as the known event for hypothesis testing. Additionally, though the precise time of first oviposition for each donor was known, the authors felt that it would not be appropriate to use this event to test the TBS method. Therefore, initial colonization was excluded as the known event for hypothesis testing. Instead, the time of placement (TOP) was chosen as it is precisely recorded for all donors placed at the ARF. Therefore, for each donor used in this study, we tested the null hypothesis that the estimated TOC or TBS = TOP.

### 2.4. Entomological TOC Estimations

As the overall objective of this study was to highlight the potential error associated with blow fly larval age estimations in cases of advanced decomposition, succession analyses were not implemented. Additionally, as two specific phenomena were examined in this study (delayed colonization and re-colonization of remains by blow flies), only larvae sampled on the first collection day for Donor 1 (i.e., the first day larvae were observed after the 115 d delay post-placement) and larvae sampled on the last collection days for Donors 2 and 3 (i.e., the observed re-colonization events) were used for TOC estimations. The age of third instar *Phormia regina* (Meigen) (Diptera: Calliphoridae) larvae (one of the most common blow fly species found at the ARF [63,64] and the only species and stage collected during these specific timepoints) was estimated using the Byrd and Allen (2001) developmental dataset [10] and standard age estimation methods [49]. A base temperature of 10 °C was chosen to calculate ADD [10] as it likely reflects the lower developmental threshold of *P. regina* [10,12]. Local temperature data for ADD calculations were obtained from the McGhee-Tyson airport weather station (Knoxville, TN) [63], located approximately 16 km from the ARF. It should be noted that although using pupae or pupal exuviae would be ideal for this type of study, pupal sampling is not performed at the ARF given the sheer number of human donors decomposing at any given time. As larvae of *P. regina* can disperse up to 26 m away from the remains during the post-feeding stage [65], it is not possible to assign any given collected pupa at the ARF to any single donor.

For each estimation to contain the full range of possible developmental durations for a given stage, the minimum ADD was calculated by summing the minimum duration recorded to complete all previous stadia, while the maximum ADD was calculated by summing the maximum duration recorded to complete all previous stadia plus the observed stadium of the specimen in question. For example, the minimum 3rd instar ADD was calculated by summing the minimum ADD required to complete the egg, 1st instar, and 2nd instar stages, while the maximum 3rd instar ADD was calculated by summing the maximum ADD required to complete the egg, 1st instar, 2nd instar, and 3rd instar stages [64]. The TOC estimations were used to estimate the TOP for each human donor in this study. A TOC estimation was considered accurate if it bracketed the TOP for the respective donor [19,64]. As TOC estimations rely on the developmental duration of each immature life stage, such estimations are limited to the maximum possible development time recorded for a given species under certain environmental conditions. For example, the maximum ADD to complete the feeding 3rd instar stage of *P. regina* has been observed as <208 ADD regardless of temperature [10], and <150 ADD for *Chrysomya rufifacies* (Macquart) (Diptera: Calliphoridae), regardless of temperature and tissue type [29].

### 2.5. Anthropological TBS Estimations

Total Body Scores (TBS) were used to calculate ADD to form an estimation of the TOP for each donor. Photographs of Donors 1 and 2 were taken twice per day until mummification led to minimal progression of decomposition, then once daily until the end of active decomposition, and once monthly until recovery of the skeleton. Photographs of Donor 3 were taken at all entomological observation timepoints. For all donors, only photographs taken from the same timepoints as those used for entomological estimations were scored for TBS. Each photograph was assessed for the extent of soft tissue decomposition via observational notes and TBS scoring [52]. Photographs were randomized and scored independently by two separate evaluators per donor. The scores were then evaluated for inter-observer error before TBS estimation accuracy was assessed. A point estimation was generated using the TBS method from Megyesi et al. [52]. A minimum and maximum ADD range was then generated using the point estimation ± standard error generated in that study (388.2 ADD). Accuracy of TBS estimations was assessed in the same manner as the entomological estimations.

## 3. Results

### 3.1. Donor 1

**Figure 1:**
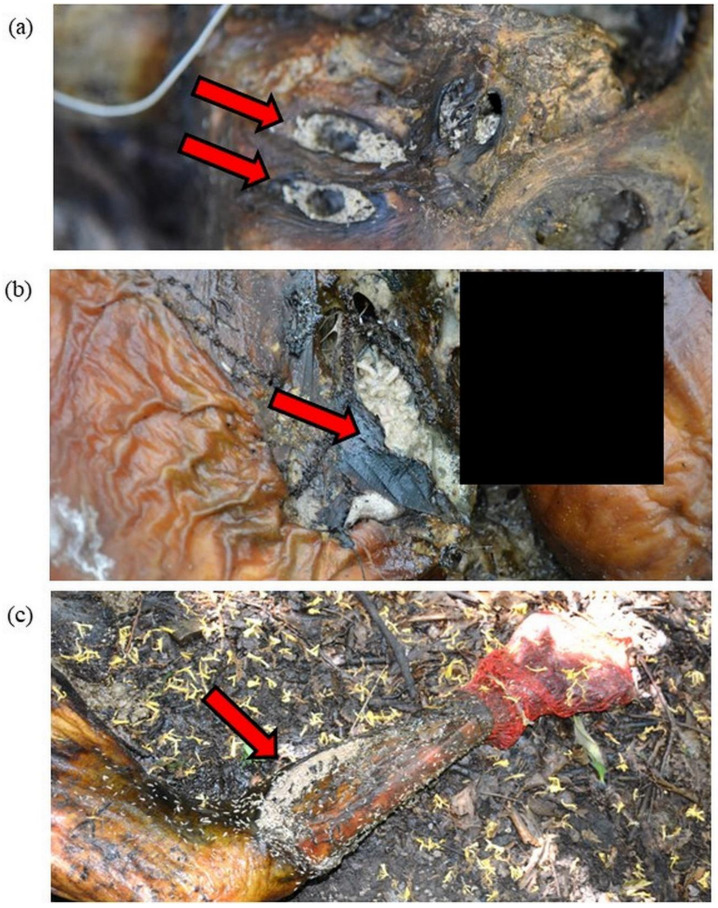
Blow fly larvae collected from each donor. (**a**) Donor 1 on the first day of larval sampling after first observation of colonization, 115 d (236.0 ADD) post-placement. Red arrows indicate larval aggregations in the eye and face. (**b**) Donor 2 on the last day of larval sampling, 280 d (1024.8 ADD) post-placement. Red arrow indicates larval aggregation at the groin. (**c**) Donor 3, 53 d (266.9 ADD) post-placement. Red arrow indicates larval aggregation on the right calf.

**Figure 2:**
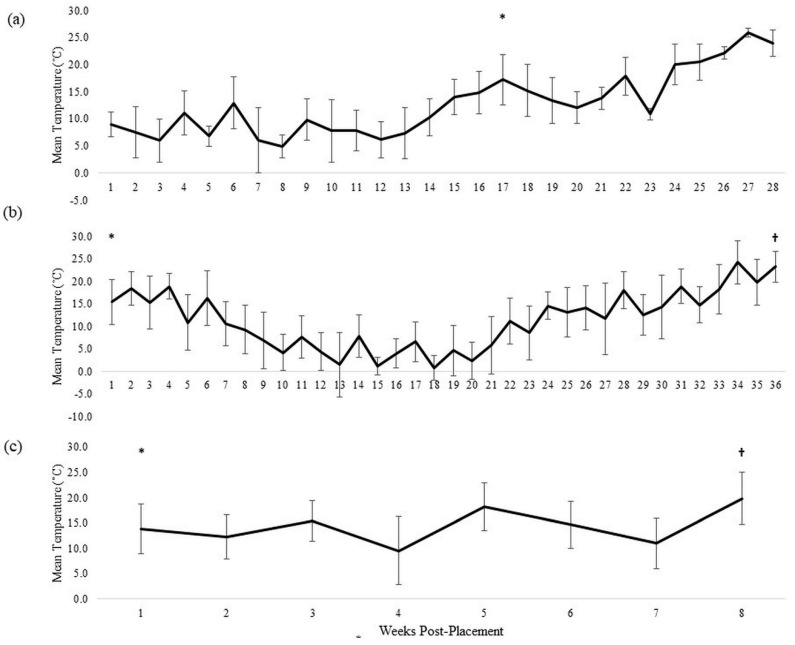
Mean (± standard deviation) temperature (in °C) for each week post-placement for each human donor used in this study. (**a**) Donor 1, (**b**) Donor 2, (**c**) Donor 3. * indicates the first observed blow fly colonization event. † indicates the first observed blow fly re-colonization event.

Donor 1 was a white female weighing 141.4 kg who died of natural causes. Placement at the ARF occurred during the late fall. Initial raccoon scavenging activity of the face (eyes were removed and mouth was manipulated) and torso (scratch marks) was recorded via game camera the night of placement. Blow flies did not colonize immediately and, though adult blow flies in the genus *Calliphora* (Robineau-Desvoidy) (Diptera: Calliphoridae) were observed near the donor 22 d (24.2 ADD) post-placement, oviposition was not observed at this time, nor were any larvae observed around this time period. This donor experienced dehydration and mummification over the next six months, and raccoon scavenging continued sporadically during this time. Increased rainfall in the spring partially rehydrated the tissue and caused mold to encompass the entire body. Purging created large pools of decomposition fluids along the ground-body interface (GBI). Adult flies returned and finally colonized Donor 1 at 115 d post-placement along the GBI at the left shoulder and face (Figure 1a). The average temperature for this time period was 9.1 ± 5.9 °C (Table 1). The third instar *P. regina* used for TOC estimations were collected from Donor 1 at 122 d post-placement (Table 2). As temperatures began to increase in early spring (Figure 2a), the head and neck were mummified with fewer than half of the skeletal elements exposed, the trunk remained distended, and purged fluids continued to pool at the GBI. The appendages exhibited brownish red and/or black coloration at the extremities and scavenger openings, and large larval aggregations were apparent at the head, neck, and groin. Donor 1 was determined to be in advanced decomposition by 191 d (835.4 ADD) post-placement based on the collapse of the abdomen, cessation of purged fluid, and drastic decrease in the observed larvae on or around the remains. At this point, the donor was unenrolled from the research project.

### 3.2. Donor 2

Donor 2 was a white female weighing 90.5 kg who died of blunt force trauma, though no external signs of trauma were observed. Placement at the ARF occurred in early autumn. The pre-CI for Donor 2 was 47.8 h, and the average temperature for this period of time was 13.3 ± 4.8 °C (Table 1). The last timepoint at which blow fly larvae were observed and collected from Donor 2 during the fall was 47 d (281.2 ADD) post-placement. At this time, the consumption phase was considered complete for this donor. However, due to prolonged decomposition (e.g., continuous fluid purge), daily observations were continued for another seven months (Figure 2b). The third instar *P. regina* used for TOC estimations were collected from Donor 2 at 280 d (1024.8 ADD) post-placement (Table 2, Figure 1b).

Raccoon scavenging (verified by game camera footage) of the limbs and the face occurred regularly during the first week after placement and continued sporadically in other areas of the body (e.g., groin) through early winter. This donor experienced bloating during mid-autumn and displayed dried mummified dermis with white mold covering the entirety of the body. The head and neck were mummified with less than half of the bone exposed, the trunk was discolored and still exhibited bloat while the appendages were moist with bone exposed in less than one half of the area being scored due to scavenging. The morphology of this individual changed little over the next eight months. Though bloat gradually recessed, this individual never exhibited a completely collapsed abdomen and continued to produce small amounts of purge fluid during this time. Additionally, a succession of various gray, white, dark green, and black molds was observed on the skin surface. The stagnation of decomposition progression and the presence of mold presented challenges for employing the TBS method (see Section 4). After blow fly re-colonization and larval activity in the early summer, the abdomen collapsed, and fluid purge ceased. The individual was unenrolled from the research project in mid-summer.

### 3.3. Donor 3

Donor 3 was a white male weighing 129.5 kg who died of natural causes. Placement at the ARF occurred in early spring. The pre-CI for Donor 3 was 95.4 h, and the average temperature for this time period was 13.6 ± 6.0 °C (Table 1). Re-colonization was observed by 44 d (214.1 ADD) post-placement (Figure 2c). The third instar *P. regina* used for TOC estimations from Donor 3 were collected at 53 d (266.9 ADD) post-placement (Figure 1c, Table 2).

Disturbance consistent with raccoon scavenging began at 8 d (34.7 ADD) post-placement, and the donor still appeared to be fresh at this time. Bloating, skin discoloration, and skin slippage of the extremities and trunk were apparent by 16 d (65.2 ADD) post-placement. Skin slippage intensified leaving a brown-orange and black dermis over the following week, and bloat with purge also continued. This individual remained in bloat until the end of the study period. The abdomen began to sag and collapse by 35 d (172.0 ADD) post-placement, while the appendages appeared leathery and dark brown. The head and neck at this time were black in color and mummified with less than half the bone exposed. By 53 d (266.9 ADD) post-placement, decomposition was considered advanced as the abdomen was fully collapsed, and no purge fluid had been present for some time. This donor was unenrolled after larval sampling on this day.

### 3.4. Accuracy of Estimation Methods

The TOC estimation for Donor 1 determined that colonization may have occurred anywhere from five weeks to five days prior to sampling, i.e., as early as four months after the true TOP. Similarly, the TOC estimation for Donor 2 determined that colonization may have occurred two weeks to two days prior to sampling, i.e., as early as almost eight months post-placement. Finally, the TOC estimation for Donor 3 determined a possible colonization window of three weeks to three days prior to sampling, which was, at the earliest, almost one month after placement. Therefore, the null hypothesis that the TOC = TOP was rejected across all donors since the TOP was not bracketed by the estimated TOC. However, as each TOP was bracketed by the ADD range produced from the anthropological TBS scores, the null hypothesis that the TBS = TOP could not be rejected across all donors.

## 4. Discussion

Entomology can be of great utility in a variety of death investigation scenarios. However, we have demonstrated that even in cases where larval blow fly evidence is plentiful, it may not be particularly useful when the longitudinal postmortem context is unknown. In this study, we presented three examples of under-reported blow fly oviposition behavior that could impact the accuracy of TOC estimations in a forensic context. Donor 1 provided an example of delayed colonization, likely due to numerous extrinsic and intrinsic variables. The combination of disease, BMI, and the cold temperatures at the time of placement likely influenced the lack of arthropod utilization of the remains for the first four months post-placement. The possibility cannot be completely ruled out that this donor could have been colonized earlier if flies selected an internal site on which to oviposit that was not visible to the observers, especially if the eggs did not hatch or larvae died shortly thereafter. However, it is unlikely that surviving larvae from such an event would have been overlooked as thorough observations of this donor were made twice daily for months. Furthermore, cold temperatures waivered around 10 °C for much of the spring (Figure 2), likely preventing most blow fly activity during this time. We hypothesize that the combination of intrinsic and extrinsic factors acted to slow the decomposition rate of this donor, which did not appear to change even when temperatures substantially increased in the late spring and summer and arthropod colonization eventually occurred. In this example, even though blow fly evidence was abundant in later decomposition, the TOC estimation was inaccurate, giving the earliest possible colonization as five weeks prior to sampling. Though the interpretation of this estimation in a forensic case is still valid (i.e., for colonization to have occurred by the estimated date, the individual was likely deceased by that time, barring myiasis in life), the fact that the earliest estimation is off by almost four months should be taken into consideration by forensic entomology practitioners who may find themselves working a case under similar conditions.

Though neither Donors 2 nor 3 experienced an unusual extension in the pre-CI, they both exemplified the phenomenon of blow fly re-colonization and resulted in inaccurate TOC estimations compared to the wider estimation ranges generated by TBS. Though the presence of immature blow flies during later states of decomposition has been documented previously, we have not found any evidence in the literature of such extreme examples as what we observed at the ARF. In a classic decomposition study using dog carcasses near Knoxville, TN, Reed [65] observed a small number of immature blow flies present during the “dry” seral stage, which he defined as a minimum of 50–90 d post-placement in the fall, spring, and summer (though this stage was left undefined for the winter) [65]. Rodriguez and Bass [66] also observed immature blow flies on human remains during the earliest portion of the “dry” decomposition stage at the ARF during the spring and summer, giving the time to reach this stage as 34 d in the spring and 13 d in the summer [66]. However, neither of these studies quantified the exact duration of immature blow flies on the remains, nor clarified if their presence was observed continuously until the dry stage or if re-colonization events may have occurred. Madra et al. [67] reported the re-colonization of pig carcasses in Poland in the second year of decomposition by the blow fly *Lucilia caesar* (Linnaeus) (Diptera: Calliphoridae), which was the only immature blow fly observed on pig carcasses in that study [67]. Anderson and VanLaerhoven [68] documented the presence of immature calliphorids on pig carcasses during the summer in British Columbia during the “advanced” stage of decomposition, which they defined as 17 to 42 d post-placement, though this is likely due to an extended larval consumption phase observed in that study [68]. Another succession study on pig carcasses in an open field habitat in New Zealand showed that immatures of *Calliphora stygia* (Fabricius) (Diptera: Calliphoridae) were present on carcasses until 77 d post-placement, though whether the sporadic occurrence of this species was due to re-colonization events or an extended larval consumption phase is not known [69]. Here, we demonstrate the phenomenon of blow fly re-colonization in Donors 2 and 3, though it was more exaggerated in Donor 2 (i.e., re-colonization occurred almost eight months post-placement and six months after cessation of the initial larval consumption phase). We again hypothesize that both intrinsic and extrinsic variables created a unique interaction that prolonged decomposition in this donor.

In the two cases of re-colonization presented here, the initial post-feeding dispersal stage was witnessed by the authors for both donors and the end of the blow fly larval consumption phase was documented (i.e., there were no more active immature blow flies present on or around the remains). Under real-world circumstances in which death and colonization can only be approximated, instances involving blow fly re-colonization of remains may never be truly known by the forensic entomologist unless artifacts (e.g., empty puparia) from the initial consumption phase are still present and detected by investigators. Given that some species (like *P. regina*) can disperse great distances from the decedent [70], it may be difficult for investigators to locate post-feeding larvae, pupae, or empty puparia even if they are present. Thus, the absence of such artifacts does not indicate the absence of a previous blow fly larval consumption or post-feeding stage. Even if such evidence were present, extensive delays in initial oviposition (as documented with Donor 1) would likely remain unknown and may not be reliably estimated by the practitioner. However, we believe that documented observations of unusual blow fly colonization activity, such as the ones presented here, are crucial for forensic entomologists to be aware of when working cases.

Within our observational study, TBS was used as an additional methodology for testing null hypotheses surrounding the TOP. Though TBS was accurate for each donor, there are several considerations anthropologists must keep in mind when implementing this method. The primary limitation of the TBS method is that it does not account for variation outside of the context in which it was designed. Environmental variables (e.g., evaluating decomposition as having a linear relationship with thermal energy does not account for seasonal change and climate variation) and other sources of soft-tissue damage not related to decomposition changes (e.g., tissue damage due to vertebrate scavenging) are not considered. Additionally, TBS assumes that decomposition is a linear process (e.g., it always progresses from fresh to skeletonization without variation). Decomposition can fluctuate with environmental conditions, and in some cases can induce regression of the remains to an earlier state of decomposition (e.g., rehydration of mummified tissues due to precipitation) [55,71], presenting obvious challenges to the practitioner.

The TBS method as presented by Megyesi et al. [52] has been assessed in temperate areas, such as South Africa and Hawaii, where the temperatures are relatively consistent throughout the seasons, yielding results that were more consistent than those from geographical regions that experience greater seasonal change [72,73]. For example, in Canada, where there is variable humidity and seasonal temperature extremes, TBS evaluation determined that no human individuals were totally skeletonized in under one year, and tissue rarely mummified [74]. This contradicts a foundational assumption of Megyesi et al. [52] who used only known PMIs of less than one year, as soft tissue was rarely present beyond this time frame in most temperate climates. In the examples given from our current study, one of the individuals retained soft tissue approaching one year after placement in the field, which led to returning cycles of rehydration and fungal growth, causing inconsistencies in TBS scores. Additionally, vertebrate animal and arthropod scavenging is also not accounted for in the original method, again, limiting its applicability. Megyesi et al. [52] elected not to consider vertebrate scavenger effects, claiming that scavenging frequently cannot be quantified in forensic cases. However, loss of flesh due to scavenging accelerates the rate of decomposition and leads to earlier skeletonization and/or a greater rate of mummification, occurrences which impede the accuracy of the TBS method [55,75]. For the donors presented in our study, skeletonization caused by scavenging resulted in higher TBS scores on average. Additionally, during retrospective scoring of photographs, changes in tissue and environmental patterns can reveal or obscure skeletal elements, again, challenging use of the method and leading to inconsistent TBS scores.

Despite these limitations, at present, TBS is the most accurate PMI estimation method available to anthropologists and, as such, continues to be used in anthropological research and casework. Although TBS methods were more accurate than entomological methods in this study, the accuracy of the TBS equation is insufficient in estimating mean ADD and requires improvement. Taken together, these studies suggest that the TBS scoring method is useable, however, the method should be amended or reconfigured to consider additional variables that impact decomposition rates.

## 5. Conclusions

The cases in this study challenge some of the basic tenets of forensic entomology. However, instead of being a condemnation of the field, this study serves to assist the forensic entomology practitioner in the interpretation of complex cases like those presented in this study. Our three examples serve as an opportunity to showcase the natural variability that can occur within the context of vertebrate decomposition ecology.

Overall, we recommend that all cases involving human remains experiencing advanced decomposition with larval blow flies present be thoroughly investigated, i.e., law enforcement, death investigators, and/or legal counsel should employ experts from relevant disciplines to interpret available evidence. This is especially true for cases in which the presence of larval blow flies is at odds with the observed soft-tissue decomposition. For example, even if arthropod evidence seems sufficient to provide a TOC estimation, an additional opinion from a forensic anthropologist trained in recognizing and interpreting soft tissue decomposition, as well as signs of vertebrate scavenging and trauma analysis, should be obtained to provide an additional estimation. Furthermore, we recommend that investigators open a line of communication between experts to better interpret the remains as a dynamic ecosystem. While immature insects may still be useful in such a scenario for age estimations (and potentially adult arthropods for succession-based estimations), their importance and utility should be interpreted with caution. At the very least, the forensic entomologist has an ethical responsibility to suggest to investigators that the opinions of relevant experts outside the field of entomology be sought out in cases where arthropod activity and decomposition seem to be at odds with one another. Finally, the authors strongly encourage the formation of collaborations between researchers in entomology, anthropology, and pathology as these disciplines intersect at the study of human decomposition [76,77]. Excluding one of these disciplines results in the loss, misinterpretation, dismissal, or total exclusion of valuable data needed to understand the dynamic processes of decomposition ecology.

## Figures and Tables

**Table 1:** Demographic information and pre-colonization data for each donor used in this study. Season_P_ = season of placement at the ARF. Season_C_ = season of initial blow fly colonization (i.e., the first oviposition event). Pre-CI = pre-colonization interval, given in hours (h). Ambient temperature of the pre-CI given as mean ± standard deviation.

Donor	Sex	Season_P_	Season_C_	Pre-CI (h)	Temp. (°C)
Donor 1	F	Winter	Spring	2760.0	9.1 ± 5.9
Donor 2	F	Fall	Fall	47.8	13.3 ± 4.8
Donor 3	M	Spring	Spring	95.4	13.6 ± 6.0

**Table 2:** Summary of third instar larval *Phormia regina* sampled on the first collection day from Donor 1 and from the last collections days for Donors 2 and 3. The total exposure time from placement to the sampling of third instar *P. regina* is given in days (d) for each donor. The 15 °C dataset from the Byrd and Allen (2001) developmental study was used to determine the time of colonization (TOC) estimation range. Temperature = mean ± standard deviation for exposure period given in °C. Site = larval sampling location on or around each donor (GBI = ground-body interface). TOP is given as the total ADD acquired during the exposure period. All TOC and total body score (TBS) estimation ranges are given in ADD utilizing a base temperature of 10 °C. * = accurate estimation.

					Estimations (ADD)	
Donor	Exposure Post-Placement (d)	Temp. (°C)	Site	TOP (ADD)	TOC	TBS
1	122	9.4 ± 6.0	Face	266.3	29.6–145.0	0.0–728.6 *
2	280	11.3 ± 8.0	Groin	1024.8	29.6–145.0	276.3–1052.7 *
3	53	14.1 ± 5.9	GBI, R. leg	266.9	29.6–145.0	19.2–795.5 *

## Data Availability

Weekly temperature data for each donor throughout decomposition are available via Dryad (https://doi.org/10.5061/dryad.ghx3ffbsd, accessed on 25 September 2022).
